# The Impact of Long COVID-19 on Mental Health: Observational 6-Month Follow-Up Study

**DOI:** 10.2196/33704

**Published:** 2022-02-24

**Authors:** Sarah Houben-Wilke, Yvonne MJ Goërtz, Jeannet M Delbressine, Anouk W Vaes, Roy Meys, Felipe VC Machado, Maarten van Herck, Chris Burtin, Rein Posthuma, Frits ME Franssen, Herman Vijlbrief, Yvonne Spies, Alex J van 't Hul, Martijn A Spruit, Daisy JA Janssen

**Affiliations:** 1 Department of Research and Education Ciro Horn Netherlands; 2 School of Nutrition and Translational Research in Metabolism Faculty of Health, Medicine and Life Sciences Maastricht University Maastricht Netherlands; 3 Department of Respiratory Medicine Maastricht University Medical Center Maastricht Netherlands; 4 REVAL Rehabilitation Research Center, BIOMED Research Institute Faculty of Rehabilitation Sciences Hasselt University Diepenbeek Belgium; 5 Lung Foundation Netherlands Amersfoort Netherlands; 6 Department of Pulmonary Disease Radboud University Medical Center Nijmegen Netherlands; 7 Care and Public Health Research Institute Faculty of Health, Medicine and Life Sciences Maastricht University Maastricht Netherlands

**Keywords:** SARS-CoV-2, corona, COVID-19, post-traumatic stress disorder, anxiety, depression, PASC

## Abstract

**Background:**

The psychological impact of COVID-19 can be substantial. However, knowledge about long-term psychological outcomes in patients with COVID-19 is scarce.

**Objective:**

In this longitudinal, observational study, we aimed to reveal symptoms of posttraumatic stress disorder (PTSD) and symptoms of anxiety and depression up to 6 months after the onset of COVID-19–related symptoms in patients with confirmed COVID-19 and persistent complaints. To demonstrate the impact in nonhospitalized patients, we further aimed to compare these outcomes between nonhospitalized and hospitalized patients.

**Methods:**

Demographics, symptoms of PTSD (Trauma Screening Questionnaire [TSQ] ≥6 points) and symptoms of anxiety and depression (Hospital Anxiety and Depression Scale [HADS] ≥8 points) were assessed at 3 and 6 months after the onset of COVID-19–related symptoms in members of online long COVID-19 peer support groups.

**Results:**

Data from 239 patients with confirmed COVID-19 (198/239, 82.8% female; median age: 50 [IQR 39-56] years) were analyzed. At the 3-month follow-up, 37.2% (89/239) of the patients had symptoms of PTSD, 35.6% (85/239) had symptoms of anxiety, and 46.9% (112/239) had symptoms of depression, which remained high at the 6-month follow-up (64/239, 26.8%, *P*=.001; 83/239, 34.7%, *P*=.90; 97/239, 40.6%, *P*=.08, respectively; versus the 3-month follow-up). TSQ scores and HADS anxiety and depression scores were strongly correlated at the 3- and 6-month follow-ups (*r*=0.63-0.71, *P*<.001). Symptoms of PTSD, anxiety, and depression were comparable between hospitalized (n=62) and nonhospitalized (n=177) patients.

**Conclusions:**

A substantial percentage of patients with confirmed COVID-19 and persistent complaints reported symptoms of PTSD, anxiety, or depression 3 and 6 months after the onset of COVID-19–related symptoms. The prevalence rates of symptoms of PTSD, anxiety, and depression were comparable between hospitalized and nonhospitalized patients and merely improved over time. Health care professionals need to be aware of these psychological complications and intervene on time in post-COVID-19 patients with persistent complaints.

**Trial Registration:**

Netherlands Trial Register NTR8705; https://www.trialregister.nl/trial/8705.

## Introduction

A traumatic event is an incident that causes physical, emotional, spiritual, or psychological harm [1]. The impact of a traumatic event varies between individuals. Most individuals are resilient, develop appropriate coping strategies, and recover without long-term consequences. Nevertheless, some experience symptoms such as unwanted upsetting memories, flashbacks, nightmares, emotional distress, or physical reactivity after exposure to traumatic reminders. If symptoms last for more than 1 month, create distress, or interfere with daily functioning, posttraumatic stress disorder (PTSD) needs to be considered [2]. PTSD has major negative consequences for patients and their families [2,3] and is associated with long-term comorbid depression and substance abuse [2], underlining the need to detect and treat the disorder.

Severe illness or acute onset of severe illness can be experienced as a traumatic event [1]. Furthermore, isolation precautions for infection prevention have been shown to be associated with severe mental health problems [4]. Accordingly, COVID-19 might also lead to PTSD. Indeed, previous studies reported prevalences of symptoms of PTSD ranging from 10% to 30% in patients discharged from the hospital [5-12]. Prevalences of symptoms of anxiety and depression in these samples vary between 5% and 42% [7,9-12] and 14% and 31% [6,9,11,12], respectively. A recently published review also confirmed the persistence of symptoms and their physical and psychosocial impact following a COVID-19 infection [13]. However, the authors mainly summarized findings from studies including hospitalized patients and concluded that more research is needed in nonhospitalized patients [13]. Indeed, the impact of so-called “mild” COVID-19 can be substantial [14-16]. To date, knowledge about psychological long-term outcomes in these patients is scarce. Mazza and colleagues [11] studied PTSD, depression, and anxiety in COVID-19 patients who visited the emergency department (ED) and compared outcomes between patients who were hospitalized or managed at home 1 month after discharge or ED visit. Psychological outcomes, including PTSD, major depression, and anxiety, were comparable between both groups or even worse in nonhospitalized patients [11]. So, even “mild” COVID-19 may not only cause symptoms of anxiety and depression but also be a stressor leading to PTSD symptoms.

As symptoms of PTSD often develop after a period of time or get worse over time [17], longer follow-up is needed. Therefore, we aimed to explore the prevalence of PTSD and symptoms of anxiety and depression at 3 and 6 months after the onset of COVID-19 symptoms and to study the association between symptoms of PTSD, anxiety, and depression in patients with confirmed COVID-19. To demonstrate the impact of COVID-19 on mental health, especially in nonhospitalized patients, we aimed to compare these outcomes between nonhospitalized and hospitalized patients. As the number of reports demonstrating a substantial burden of psychological trauma in patients with less severe COVID-19 is increasing [18], we hypothesized in advance that the long-term impact of COVID-19 on mental health, even in nonhospitalized patients, is substantial.

## Methods

### Study Design, Setting, and Participants

Members from 2 Facebook groups for coronavirus patients with persistent complaints in The Netherlands (~11,000 members; “Corona ervaringen en langdurige klachten!”) [19] and Flanders (Belgium, ~1200 members; “Corona patiënten met langdurige klachten (Vlaanderen)”) [20] as well as ~1200 people who registered on the website of the Lung Foundation Netherlands (Coronaplein [21]) were invited to complete an online open survey between June 4, 2020 and June 11, 2020 and between August 31, 2020 and September 8, 2020. In total, 1939 members completed the first survey, of which 1556 consented to be approached for future research (see Multimedia Appendix 1).

### Ethical Approval

The medical ethics committee of Maastricht University stated that the Medical Research Involving Human Subjects Act (WMO) did not apply for this study and that an official approval of this study by the committee was not required (METC2020-1978 and METC2020-2554). The medical ethics committee of Hasselt University formally judged and approved the study (MEC2020/041). All respondents gave digital informed consent at the start of both surveys. Without informed consent, the survey could not be continued. The study was registered in the Dutch trial register. Health status, care dependency, and other characteristics of this study sample have been described elsewhere [14-16,22-25].

### Clinical Characteristics

Demographics (age, gender), marital status (yes, not married, or living with partner), educational level (low, middle, or high), BMI, and self-reported pre-existing comorbidities were assessed. Furthermore, the survey contained questions regarding the diagnosis of COVID-19: self-reported, polymerase chain reaction (PCR)-confirmed, and/or radiologic confirmed. A confirmed diagnosis was based on a computed tomography (CT) scan or reverse transcription PCR (RT-PCR). Self-reported health status (good, moderate, or poor), symptoms during the infection and during follow-up, as well as the date of the onset of these COVID-19 symptoms were assessed as previously described [15,23].

### PTSD

The Trauma Screening Questionnaire (TSQ) was used as a screening instrument for PTSD [26]. The TSQ is a 10-item self-report scale consisting of 5 re-experiencing items and 5 arousal items from the Diagnostic and Statistical Manual of Mental Disorders IV (DSM-IV) criteria for PTSD.

Re-experiencing items include (1) upsetting thoughts or memories about the event that have come into your mind against your will, (2) upsetting dreams about the event, (3) acting or feeling as though the event was happening again, (4) feeling upset by reminders of the event, and (5) bodily reactions (such as fast heartbeat, stomach churning, sweatiness, dizziness) when reminded of the event.

Arousal items include (1) difficulty falling or staying asleep, (2) irritability or outbursts of anger, (3) difficulty concentrating, (4) heightened awareness of potential dangers to yourself and others, and (5) feeling jumpy or being startled at something unexpected.

For the current study, the event was defined as a “corona infection.” Participants were asked whether they experienced each symptom at least twice in the past week. In total, 10 questions could be answered with yes or no. A cut-off score of ≥6 points was used to identify patients at risk of having PTSD [26].

### Anxiety and Depression

Symptoms of anxiety and depression were assessed using the Hospital Anxiety and Depression scale (HADS), which is divided into an anxiety subscale and a depression subscale [27]. Total scores for each subscale range from 0 (optimal) to 21 points (worst). A cut-off score of ≥8 points was used to identify the presence of clinically relevant symptoms of anxiety or depression [27,28].

### Statistical Analyses

Continuous data are presented as mean (SD) or median (IQR), as appropriate. Categorical data are presented as absolute and relative frequencies.

Differences between 3- and 6-month follow-ups were evaluated with the McNemar test, paired sample *t* test, or Wilcoxon signed rank test, as appropriate. Differences between hospitalized (without admission to the intensive care unit) and nonhospitalized patients were tested with chi square tests, independent sample *t* tests, or Mann-Whitney *U* tests. Correlations between TSQ and HADS anxiety and depression scores were assessed with scatterplots and Spearman rho. Correlation coefficients of 0.00-0.19, 0.20-0.39, 0.40-0.59, 0.60-0.79, and 0.80-1.00 were defined as very weak, weak, moderate, strong, or very strong, respectively [29]. Statistics were performed using SPSS version 25.0. A priori, the level of significance was set at *P*<.05.

## Results

### Survey Completion

Of the 1556 patients who completed the first survey about 3 months after the onset of COVID-19–related symptoms and consented to be approached for future research, 1005 patients (65%) completed the second survey about 6 months after the onset of COVID-19–related symptoms (see Multimedia Appendix 1). For the current study, only patients who completed both surveys and who had a confirmed diagnosis based on CT/RT-PCR were included for analyses (n=239). Results from patients with suspected COVID-19 (ie, patients who did not have a formal COVID-19 test at the time of the suspected infection) who completed both surveys (n=766) are presented online and show similarities to those of patients with a confirmed COVID-19 diagnosis (see Multimedia Appendix 2). Time between symptom onset and completion of the questionnaire was 10.4 (2.4) weeks for the first survey and 22.6 (2.4) weeks for the second survey. As demonstrated in another analysis of the current data [23], the results of the first survey were comparable between patients who did and did not complete the second survey.

### Patient Characteristics

Generally, patients were middle-aged women. The majority of patients were married or living with a partner (173/239, 72.4%) and had no pre-existing comorbidities (142/239, 59.4%). Self-reported health status was generally good before the infection, which was significantly worse 3 months after infection and during the 6 months of follow-up. During the infection, patients reported a median of 15 (IQR 11-18) different symptoms, which decreased to medians of 6 (IQR 4-9) and 6 (IQR 3-8) symptoms at the 3- and 6-month follow-ups, respectively (Table 1).

**Table 1:** Patient characteristics.

Characteristics			All patients (n=239)		Hospitalized patients (n=62)		Nonhospitalized patients (n=177)		*P* value^a^
Women, n (%)			198 (82.8)		39 (62.9)		159 (89.8)		<.001
Age (years), median (IQR)			50.0 (39.0-56.0)		53.0 (47.8-60.0)		48.0 (37.5-54.5)		<.001
BMI (kg/m^2^), median (IQR)			26.0 (23.4-30.5)		28.2 (24.8-32.6)		25.6 (23.0-29.4)		.005
Married/living with partner, n (%)			173 (72.4)		43 (69.4)		130 (73.4)		.51
**Educational level, n (%)**									
	Low	6 (2.5)		6 (9.7)		0 (0)		<.001	
	Medium	126 (52.7)		32 (51.6)		94 (53.1)		.84	
	High	107 (44.8)		24 (38.7)		83 (46.9)		.27	
**Pre-existing comorbidities, n (%)**									
	None	142 (59.4)		28 (45.2)		114 (64.4)		.008	
	1	62 (25.9)		23 (37.1)		39 (22.0)		.02	
	≥2	35 (14.6)		11 (17.7)		24 (13.6)		.42	
**Good self-reported health status before infection, n (%)**									
	Before infection	208 (87.0)		49 (79.0)		159 (89.8)		.03	
	After 3 months	22 (9.2)		10 (16.1)		12 (6.8)		.03	
	After 6 months	40 (16.7)		12 (19.4)		28 (15.8)		.52	
**Number of symptoms, median (IQR)**									
	During infection	15 (11-18)		14 (10-17)		15 (12-18)		.20	
	After 3 months	6 (4-9)		6 (4-8)		6 (4-9)		.32	
	After 6 months	6 (3-8)		6 (2-8)		6 (3-8)		.62	

^a^Hospitalized patients compared with nonhospitalized patients.

### Symptoms of PTSD

At 3 months after the onset of symptoms, 37.2% (89/239) of the patients were at risk for PTSD, which decreased to 26.8% (64/239) at the 6-month follow-up (*P*=.001; Table 2).

**Figure 1:**
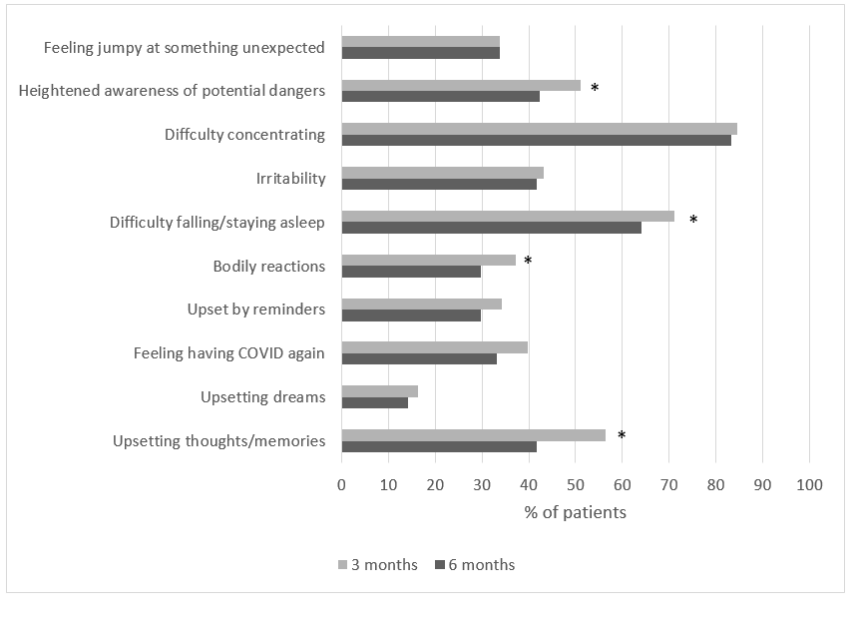
Percentage of patients reporting each item on the Trauma Screening Questionnaire (TSQ) at 3 and 6 months after the onset of COVID-19 symptoms (n=239). *P≤0.05 3 months versus 6 months.

At 3 months after the onset of symptoms, patients most frequently experienced problems with “difficulty concentrating” (202/239, 84.5%), “difficulty falling or staying asleep” (170/239, 71.1%), and “upsetting thoughts or memories about the event that have come into your mind against your will” (135/239, 56.5%). Of the TSQ items, 4 improved significantly over time (see Figure 1 for details).

**Table 2:** Symptoms of posttraumatic stress disorder, anxiety, and depression.

Assessments		All patients (n=239)	*P* value^a^	Hospitalized patients (n=62)	*P* value^a^	Nonhospitalized patients (n=177)	*P* value^a^	*P* value^b^
**Trauma Screening Questionnaire**								
	Total score after 3 months, mean (SD)	4.7 (2.5)	<.001	4.7 (2.9)	.02	4.7 (2.4)	0.001	.83
	Total score after 6 months, mean (SD)	4.1 (2.5)		4.2 (2.8)		4.1 (2.4)		.89
	Total score ≥6 points after 3 months, n (%)	89 (37.2)	.001	27 (43.5)	.02	62 (35.0)	.02	.23
	Total score ≥6 points after 6 months, n (%)	64 (26.8)		19 (30.6)		45 (25.4)		.42
**HADS^c^ anxiety subscale**								
	Total score after 3 months, mean (SD)	6.5 (4.0)	.15	6.5 (4.5)	.06	6.5 (3.8)	.51	.92
	Total score after 6 months, mean (SD)	6.2 (4.0)		5.7 (4.3)		6.3 (3.9)		.31
	Total score ≥8 after 3 months, n (%)	85 (35.6)	.90	21 (33.9)	.55	64 (36.2)	.99	.75
	Total score ≥8 after 6 months, n (%)	83 (34.7)		18 (29.0)		65 (36.7)		.27
**HADS depression subscale**								
	Total score after 3 months, mean (SD)	7.2 (4.0)	.001	7.0 (4.1)	.19	7.3 (3.9)	.003	.59
	Total score after 6 months, mean (SD)	6.5 (3.0)		6.4 (4.6)		6.6 (3.6)		.83
	Total score ≥8 after 3 months, n (%)	112 (46.9)	.08	31 (50.0)	.33	81 (45.8)	.19	.57
	Total score ≥8 after 6 months, n (%)	97 (40.6)		26 (41.9)		71 (40.1)		.80

^a^3 months compared with 6 months.

^b^Hospitalized patients compared with nonhospitalized patients.

^c^HADS: Hospital Anxiety and Depression Scale.

### Symptoms of Anxiety and Depression

Clinically relevant symptoms of anxiety and depression were detected in 35.6% (85/239) and 46.9% (112/239), respectively, of all patients at the 3-month follow-up. The prevalence of symptoms of anxiety and depression remained high at the 6-month follow-up (83/239, 34.7% for symptoms of anxiety, *P*=.90; 97/239, 40.6% for symptoms of depression, *P*=.08; Table 2).

### Associations Between Symptoms

**Figure 2:**
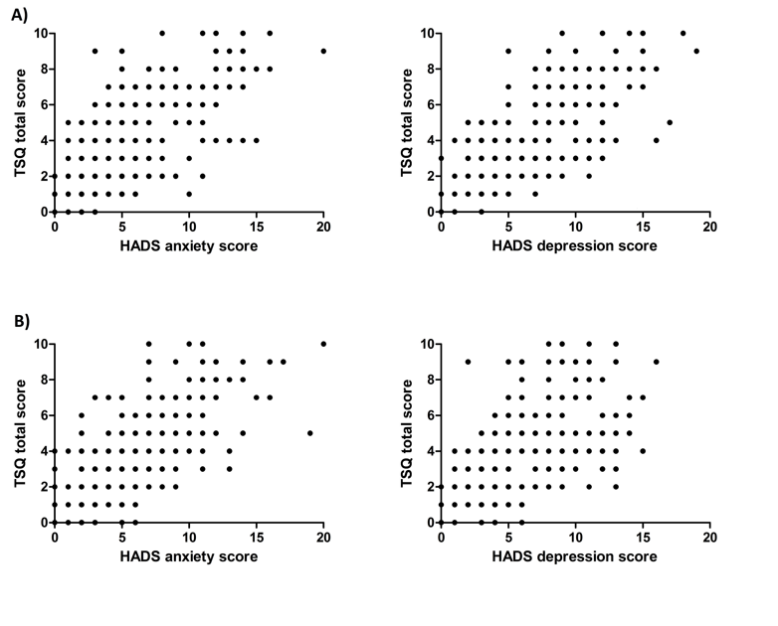
Correlations between Trauma Screening Questionnaire (TSQ) scores and Hospital Anxiety and Depression Scale (HADS) scores at (A) 3 and (B) 6 months after the onset of COVID-19–related symptoms.

TSQ scores and HADS anxiety and depression scores were strongly correlated at the 3- and 6-month follow-ups (*P*=0.631-0.714, *P*≤.001; Figure 2).

Of those patients who were at risk for PTSD at 3- and 6-month follow-ups, two-thirds had clinically relevant symptoms of anxiety and depression.

### Differences Between Hospitalized and Nonhospitalized Patients

Compared with hospitalized patients (n=62), nonhospitalized patients (n=177) more often were women, were younger, had a lower BMI, and reported fewer pre-existing comorbidities. Nonhospitalized patients more often reported good health status before the infection, while more hospitalized patients reported good health status at the 3-month follow-up (Table 1). The prevalences of symptoms of PTSD were comparable between hospitalized and nonhospitalized patients (3-month follow-up: 27/62, 43.5% vs 62/127, 35.0%, *P*=.23; 6-month follow-up: 19/62, 30.6% vs 45/127, 25.4%, *P*=.42; Table 2). Prevalence distributions for the separate TSQ items at 3 and 6 months were generally comparable between hospitalized and nonhospitalized patients, except for “upsetting dreams about the event” (*P*=.04) and “feeling upset by reminders of the event” (*P*=.03), which were more prominent in hospitalized patients at the 6-month follow-up, and “irritability or outbursts of anger” (*P*=.04), which was more prominent in nonhospitalized patients at the 6-month follow-up (see Multimedia Appendix 3). Nonhospitalized patients reported a similar level of symptoms of anxiety and depression compared with hospitalized patients at the 3- and 6-month follow-ups (Table 2).

### Patients With Suspected COVID-19

The results of the 766 patients with suspected COVID-19 are comparable to those of patients with a confirmed COVID-19 diagnosis (see Multimedia Appendix 4 and Multimedia Appendix 5).

## Discussion

### Principal Findings

A relevant percentage of nonhospitalized patients were at risk of PTSD and had clinically relevant symptoms of anxiety and depression at 3 and 6 months after the onset of COVID-19–related symptoms. The prevalence rates of symptoms of PTSD, anxiety, and depression were comparable between hospitalized and nonhospitalized patients and merely improved over time. A previous analysis of the current data demonstrated that 95% of the patients still experience 1 or more symptoms at the 6-month follow-up, which can affect work productivity, functional status, and quality of life [23]. This study indicates the long-term impact of COVID-19 on mental health, especially in nonhospitalized patients with COVID-19 with persistent complaints.

### Comparison With Prior Studies

This study supports the suggestion by Gu and colleagues [30] that the impact of COVID-19 is no less than the impact of other stressors (eg, natural disasters, technological accidents). The prevalence of patients at risk of PTSD (89/239, 37.2%) is nearly comparable with the prevalence (41%) found in 76 victims of violence and traffic accidents [31]. In addition, a meta-analysis suggested that around 20% of adult critical care survivors experience PTSD [32], while in previous coronavirus pandemics (SARS and MERS outbreaks), PTSD occurred in 32.2% of individuals who recovered from coronavirus infection [33]. This demonstrates the long-term impact of severe viral disease. Whether PTSD is also highly prevalent in the aftermath of different COVID-19 populations remains to be determined. Previous published studies reported prevalences of symptoms of PTSD ranging from 10% to 30% in patients discharged from the hospital [5-12], which is lower than the current prevalence rates from hospitalized as well as nonhospitalized patients. Hellemons and colleagues [12] reported an even lower prevalence for PTSD (4%) in patients with COVID-19 6 months after hospital discharge. With regards to anxiety and depression, a meta-analysis demonstrated pooled prevalences of 47% and 45% for anxiety and depression, respectively, in patients with COVID-19 [34]. In addition, Kong and colleagues [35] used the same assessment method (HADS) and the same cut-off score (≥8 points) as our study to evaluate symptoms of anxiety and depression in hospitalized patients with COVID-19 and showed prevalences of 35% and 29%, respectively. This study reported a comparable prevalence of anxiety symptoms, though a higher prevalence of depression symptoms, which might be explained by the fact that our study included patients with long COVID-19 symptoms. This study showed that symptoms of PTSD, anxiety, and depression merely improved over time. This is in line with a recent study demonstrating that anxiety and PTSD did not change between 1- and 3-month follow-ups in COVID-19 survivors after hospital discharge [10]. Our paper supports the authors’ conclusion that patient follow-up is an essential component of disease management [10], especially in patients with persistent complaints.

### Reasoning Behind Similar Outcomes Between Hospitalized and Nonhospitalized Patients

Notably, the impact of long COVID-19 on mental health was comparable between hospitalized and nonhospitalized patients, which might, in part, be explained by the following causes.

#### Lack of Care

Although the number of symptoms during the acute phase of the infection was comparable, hospitalized patients might have experienced a more severe clinical cause and manifestations of the disease. However, they probably learned to cope with the persistent symptoms of the disease during hospital admission or received aftercare immediately after discharge. Indeed, compared with nonhospitalized patients, hospitalized patients more often received any care from a physiotherapist, medical specialist (eg, pulmonologist), psychologist, dietician, or nurse during or after the onset of symptoms [15]. Moreover, not only during the infection but also in the months following the onset of symptoms, hospitalized patients received physiotherapy or rehabilitation substantially more often than nonhospitalized patients [23]. This might have contributed to the higher percentage of hospitalized patients reporting good self-reported health during the follow-up compared with nonhospitalized patients.

#### Unmet Needs

A previous analysis of the current data demonstrated that patients perceive a broad variety of unmet care and information needs, perceive lack of support and understanding from family members, and are worried about incomplete recovery [24]. They indicated the need for certainty and wanted clear information [24], which might further contribute to anxious or depressive feelings or add to “irritability” and “feeling jumpy or being startled at something unexpected.” Indeed, in uncertain or frightening situations, clear and precise information is essential [36]. Furthermore, lacking knowledge of the pandemic and less family support were associated with increased risk of depression or anxiety [37].

In addition, patients indicated the need for a test to confirm their diagnosis or certainty that they have had COVID-19: “Without a test, it’s all in your mind, it’s a psychological thing” [24]. This study demonstrated once again the (mental) impact on patients with suspected COVID-19. We therefore underline the importance of also considering these patients for relevant interventions.

#### Social Isolation

The impact of COVID-19 on mental health is tremendous, not only in patients who have been infected with the disease: “Humans are social beings; hence, it is not surprising that extended periods of social isolation are so difficult to cope with” [38]. Being in lockdown can lead to feelings of confinement and loneliness [39]. Loneliness has been described as one of the greatest threats to our health, survival, and well-being [36]. Furthermore, besides the physical harm of COVID-19, periods of confinement can be associated with depression, anxiety, and PTSD [38]. Quarantine has been associated with increased rates of suicide, anger, acute stress disorder, depression, and PTSD, with symptoms continuing even years after quarantine ends [38].

### Methodological Considerations

To the best of our knowledge, this study is one of the first investigating the mental impact of patients with confirmed COVID-19 and persistent complaints at 3- and 6-month follow-ups after the onset of COVID-19–related symptoms. In the following paragraphs, several strengths and limitations are discussed.

First, the majority of the study sample was middle-aged women, which may limit the external validity of the study. However, this needs to be discussed in a broader context: During the COVID-19 pandemic, female gender has been shown to be associated with an increased risk of anxiety or depression as well as PTSD [37], and women and younger age groups were at risk of poorer mental health [40,41]. Additionally, older age was associated with better mental health (ie, lower levels of anxiety and depression). However, the role of age during the recovery period is not known yet [42]. Nonetheless, the gender distribution is consistent with previous studies [43-47] and can partly be explained by the higher number of women in COVID-19 support groups [44,45]. Moreover, it has been suggested that long COVID is more common in women than in men [47]. Second, a comprehensive picture of the mental health status of the study sample before infection is not known. We do know that patients with psychiatric history were more likely to have PTSD as demonstrated by Kang and colleagues [48]. However, of those 239 confirmed cases included in our study, 2 (0.8%) patients reported that they had been treated for anxiety, while 5 (2.1%) patients reported that they had been treated for depressive mood before infection (see Multimedia Appendix 6), suggesting a limited influence of pre-existing mental health problems in this study. Third, a longer follow-up period might be relevant to detect even more patients with PTSD symptoms, as there can be a delay of months or even years before symptoms appear for some people [49]. Fourth, there is lack of data about other COVID-19–related stressors (such as death or serious illness [due to COVID-19] of a loved one, social isolation, or loss of work), which might have influenced mental health status. Finally, since the study sample consisted of selected participants recruited through online platforms and specifically targeted patients with persistent complaints, the external validity of our findings is limited.

Taking these considerations into account, this study sample still represents an important, as well as increasing, group of patients with a rising demand for health care services [18].

### Recommendations

Patients with persistent complaints report clinically relevant symptoms of anxiety, depression, and PTSD, supporting the urgent call for rapid response to the mental health impacts of COVID-19 [36], not only for patients but also for the general population [50]. Health care professionals need to be aware that patients, especially women and younger age groups [40,41], are at risk of developing such symptoms and need to intervene on time (eg, prevention of onset of these symptoms during the acute phase or treatment and prevention of progression during follow-up). General practitioners and occupational physicians play a central role in the management of mental disorders during the COVID-19 pandemic, involving early detection, (risk) assessment, and referral [51-53]. Accordingly, psychological-behavioral interventions might be provided if needed, which has been shown to reduce symptoms of anxiety and depression in patients with COVID-19 [35]. Internet-based cognitive and behavioral therapy has been shown to be an effective and acceptable alternative to therapist-delivered treatments for anxiety and depression, while the efficacy for treating PTSD is uncertain [54]. Systemic and well-designed intervention trials with robust outcome evaluations are needed to reveal strategies and models of prevention for PTSD among individuals affected by epidemics of other infectious diseases, such as COVID-19 [50]. Additionally, creating opportunities for patients to access platforms and care team members through telehealth is recommended [55]. Finally, as the experienced distress is a normal human response to a serious crisis, it is therefore advised to recognize and accept these feelings to prevent them from turning into a disorder [36].

### Conclusion

This study shows that a substantial percentage of hospitalized as well as nonhospitalized patients with persistent complaints after COVID-19 has clinically relevant symptoms of PTSD, anxiety, and depression. These symptoms were present at 3 months after the onset of COVID-19–related symptoms and remained high at the 6-month follow-up. Health care professionals as well as patients need to be aware of these symptoms and intervene on time.

## Appendix

Multimedia Appendix 1 Study flow chart.

Multimedia Appendix 2 Characteristics of patients with presumed COVID-19.

Multimedia Appendix 3 Percentage of (a) hospitalized and (b) nonhospitalized patients for separate Trauma Screening Questionnaire (TSQ) items 3 and 6 months after the onset of COVID-19 symptoms.

Multimedia Appendix 4 Symptoms of post-traumatic stress disorder, anxiety, and depression.

Multimedia Appendix 5 Percentage of patients for separate Trauma Screening Questionnaire (TSQ) items 3 and 6 months after the onset of COVID-19 symptoms (patients with suspected COVID-19, n=766).

Multimedia Appendix 6 Self-reported pre-existing comorbidities.

## Abbreviations

CT: computed tomography
DSM-IV: Diagnostic and Statistical Manual of Mental Disorders
ED: emergency department
HADS: Hospital Anxiety and Depression Scale
PTSD: posttraumatic stress disorder
RT-PCR: reverse transcription polymerase chain reaction
TSQ: Trauma Screening Questionnaire
WMO: Medical Research Involving Human Subjects Act

## References

[1] Traumatic Events. Healthline.

[2] (2014). Trauma-Informed Care in Behavioral Health Services. Center for Substance Abuse Treatment.

[3] Wintermann G, Petrowski K, Weidner K, Strauß B, Rosendahl J (2019). Impact of post-traumatic stress symptoms on the health-related quality of life in a cohort study with chronically critically ill patients and their partners: age matters. Crit Care.

[4] Hossain MM, Sultana A, Purohit N (2020). Mental health outcomes of quarantine and isolation for infection prevention: a systematic umbrella review of the global evidence. Epidemiol Health.

[5] Chang MC, Park D (2020). Incidence of post-traumatic stress disorder after coronavirus disease. Healthcare (Basel).

[6] Naidu SB, Shah AJ, Saigal A, Smith C, Brill SE, Goldring J, Hurst JR, Jarvis H, Lipman M, Mandal S (2021). The high mental health burden of "Long COVID" and its association with on-going physical and respiratory symptoms in all adults discharged from hospital. Eur Respir J.

[7] Janiri D, Carfì A, Kotzalidis GD, Bernabei R, Landi F, Sani G, Gemelli Against COVID-19 Post-Acute Care Study Group (2021). Posttraumatic stress disorder in patients after severe COVID-19 infection. JAMA Psychiatry.

[8] Tarsitani L, Vassalini P, Koukopoulos A, Borrazzo C, Alessi F, Di Nicolantonio C, Serra R, Alessandri F, Ceccarelli G, Mastroianni CM, d'Ettorre G (2021). Post-traumatic stress disorder among COVID-19 survivors at 3-month follow-up after hospital discharge. J Gen Intern Med.

[9] de Graaf MA, Antoni ML, Ter Kuile MM, Arbous MS, Duinisveld AJF, Feltkamp MCW, Groeneveld GH, Hinnen SCH, Janssen VR, Lijfering WM, Omara S, Postmus PE, Ramai SRS, Rius-Ottenheim N, Schalij MJ, Schiemanck SK, Smid L, Stöger J L, Visser LG, de Vries JJC, Wijngaarden MA, Geelhoed JJM, Roukens AHE (2021). Short-term outpatient follow-up of COVID-19 patients: A multidisciplinary approach. EClinicalMedicine.

[10] DE Lorenzo R, Cinel E, Cilla M, Compagnone N, Ferrante M, Falbo E, Patrizi A, Castellani J, Magnaghi C, Calvisi SL, Arcidiacono T, Lanzani CL, Canti V, Mazza MG, Martinenghi S, Vitali G, Benedetti F, Ciceri F, Conte C, Rovere Querini P (2021). Physical and psychological sequelae at three months after acute illness in COVID-19 survivors. Panminerva Med.

[11] Mazza MG, De Lorenzo R, Conte C, Poletti S, Vai B, Bollettini I, Melloni EMT, Furlan R, Ciceri F, Rovere-Querini P, Benedetti F, COVID-19 BioB Outpatient Clinic Study group (2020). Anxiety and depression in COVID-19 survivors: Role of inflammatory and clinical predictors. Brain Behav Immun.

[12] Hellemons ME, Huijts S, Bek L, Berentschot J, Nakshbandi G, Schurink CAM, Vlake J, van Genderen ME, van Bommel J, Gommers D, Odink A, Ciet P, Shamier MC, GeurtsvanKessel C, Baart SJ, Ribbers GM, van den Berg-Emons HG, Heijenbrok-Kal MH, Aerts JGJV (2021). Persistent health problems beyond pulmonary recovery up to 6 months after hospitalization for SARS-CoV-2; a longitudinal study of respiratory, physical and psychological outcomes. Ann Am Thorac Soc.

[13] Michelen M, Manoharan L, Elkheir N, Cheng V, Dagens D, Hastie C, O'Hara M, Suett JC, Dahmash D, Bugaeva P, Rigby I, Munblit D, Harriss E, Burls A, Foote C, Scott J, Carson G, Olliaro P, Sigfrid L, Stavropoulou C (2021). Characterising long COVID: a living systematic review. BMJ Glob Health.

[14] Vaes AW, Machado FVC, Meys R, Delbressine JM, Goertz YMJ, Van Herck M, Houben-Wilke S, Franssen FME, Vijlbrief H, Spies Y, Van 't Hul AJ, Burtin C, Janssen DJA, Spruit MA (2020). Care dependency in non-hospitalized patients with COVID-19. J Clin Med.

[15] Goërtz YMJ, Van Herck M, Delbressine JM, Vaes AW, Meys R, Machado FVC, Houben-Wilke S, Burtin C, Posthuma R, Franssen FME, van Loon N, Hajian B, Spies Y, Vijlbrief H, van 't Hul AJ, Janssen DJA, Spruit MA (2020). Persistent symptoms 3 months after a SARS-CoV-2 infection: the post-COVID-19 syndrome?. ERJ Open Res.

[16] Meys R, Delbressine JM, Goërtz YMJ, Vaes AW, Machado FVC, Van Herck M, Burtin C, Posthuma R, Spaetgens B, Franssen FME, Spies Y, Vijlbrief H, Van't Hul AJ, Janssen DJA, Spruit MA, Houben-Wilke S (2020). Generic and respiratory-specific quality of life in non-hospitalized patients with COVID-19. J Clin Med.

[17] McFarlane AC (2010). The long-term costs of traumatic stress: intertwined physical and psychological consequences. World Psychiatry.

[18] Badenoch J, Cross B, Hafeez D, Song J, Watson C, Butler M, Nicholson TR, Rooney AG, (The SARS-COV-neuro collaboration) (2020). Post-traumatic symptoms after COVID-19 may (or may not) reflect disease severity. Psychol Med.

[19] Corona ervaringen en langdurige klachten!. Facebook.

[20] Corona patiënten met langdurige klachten (Vlaanderen). Facebook.

[21] Lung Foundation Netherlands. Coronaplein.

[22] Machado FVC, Meys R, Delbressine JM, Vaes AW, Goërtz YMJ, van Herck M, Houben-Wilke S, Boon GJAM, Barco S, Burtin C, van 't Hul A, Posthuma R, Franssen FME, Spies Y, Vijlbrief H, Pitta F, Rezek SA, Janssen DJA, Siegerink B, Klok FA, Spruit MA (2021). Construct validity of the Post-COVID-19 Functional Status Scale in adult subjects with COVID-19. Health Qual Life Outcomes.

[23] Vaes AW, Goërtz YMJ, Van Herck M, Machado FVC, Meys R, Delbressine JM, Houben-Wilke S, Gaffron S, Maier D, Burtin C, Posthuma R, van Loon NPH, Franssen FME, Hajian B, Simons SO, van Boven JFM, Klok FA, Spaetgens B, Pinxt CMH, Liu LYL, Wesseling G, Spies Y, Vijlbrief H, van 't Hul AJ, Janssen DJA, Spruit MA (2021). Recovery from COVID-19: a sprint or marathon? 6-month follow-up data from online long COVID-19 support group members. ERJ Open Res.

[24] Houben-Wilke S, Delbressine J, Vaes A, Goërtz YMJ, Meys R, Machado F, Van Herck M, Burtin C, Posthuma R, Franssen FM, van Loon NHP, Hajian B, Vijlbrief H, Spies Y, van 't Hul A, Janssen DJ, Spruit MA (2021). Understanding and being understood: information and care needs of 2113 patients with confirmed or suspected COVID-19. J Patient Exp.

[25] Delbressine JM, Machado FVC, Goërtz YMJ, Van Herck M, Meys R, Houben-Wilke S, Burtin C, Franssen FME, Spies Y, Vijlbrief H, van 't Hul AJ, Janssen DJA, Spruit MA, Vaes AW (2021). The impact of post-COVID-19 syndrome on self-reported physical activity. Int J Environ Res Public Health.

[26] Brewin CR, Rose S, Andrews B, Green J, Tata P, McEvedy C, Turner S, Foa EB (2002). Brief screening instrument for post-traumatic stress disorder. Br J Psychiatry.

[27] Zigmond AS, Snaith RP (1983). The hospital anxiety and depression scale. Acta Psychiatr Scand.

[28] Olssøn I, Mykletun A, Dahl AA (2005). The Hospital Anxiety and Depression Rating Scale: a cross-sectional study of psychometrics and case finding abilities in general practice. BMC Psychiatry.

[29] Swinscow TDV (1997). Correlation and regression. Statistics at Square One.

[30] Gu Y, Zhu Y, Xu F, Xi J, Xu G (2021). Factors associated with mental health outcomes among patients with COVID-19 treated in the Fangcang shelter hospital in China. Asia Pac Psychiatry.

[31] Dekkers AMM, Olff M, Näring GWB (2010). Identifying persons at risk for PTSD after trauma with TSQ in the Netherlands. Community Ment Health J.

[32] Righy C, Rosa RG, da Silva RTA, Kochhann R, Migliavaca CB, Robinson CC, Teche SP, Teixeira C, Bozza FA, Falavigna M (2019). Prevalence of post-traumatic stress disorder symptoms in adult critical care survivors: a systematic review and meta-analysis. Crit Care.

[33] Rogers JP, Chesney E, Oliver D, Pollak TA, McGuire P, Fusar-Poli P, Zandi MS, Lewis G, David AS (2020). Psychiatric and neuropsychiatric presentations associated with severe coronavirus infections: a systematic review and meta-analysis with comparison to the COVID-19 pandemic. The Lancet Psychiatry.

[34] Deng J, Zhou F, Hou W, Silver Z, Wong CY, Chang O, Huang E, Zuo QK (2021). The prevalence of depression, anxiety, and sleep disturbances in COVID-19 patients: a meta-analysis. Ann N Y Acad Sci.

[35] Kong X, Kong F, Zheng K, Tang M, Chen Y, Zhou J, Li Y, Diao L, Wu S, Jiao P, Su T, Dong Y (2020). Effect of psychological-behavioral intervention on the depression and anxiety of COVID-19 patients. Front Psychiatry.

[36] Pietrabissa G, Simpson SG (2020). Psychological consequences of social isolation during COVID-19 outbreak. Front Psychol.

[37] Vindegaard N, Benros ME (2020). COVID-19 pandemic and mental health consequences: Systematic review of the current evidence. Brain Behav Immun.

[38] Jurblum M, Ng CH, Castle DJ (2020). Psychological consequences of social isolation and quarantine: Issues related to COVID-19 restrictions. Aust J Gen Pract.

[39] Burn W, Mudholkar S (2020). Impact of COVID-19 on mental health: Update from the United Kingdom. Indian J Psychiatry.

[40] Li LZ, Wang S (2020). Prevalence and predictors of general psychiatric disorders and loneliness during COVID-19 in the United Kingdom. Psychiatry Res.

[41] Smith L, Jacob L, Yakkundi A, McDermott D, Armstrong NC, Barnett Y, López-Sánchez GF, Martin S, Butler L, Tully MA (2020). Correlates of symptoms of anxiety and depression and mental wellbeing associated with COVID-19: a cross-sectional study of UK-based respondents. Psychiatry Res.

[42] Wilson JM, Lee J, Shook NJ (2021). COVID-19 worries and mental health: the moderating effect of age. Aging Ment Health.

[43] Davido B, Seang S, Tubiana R, de Truchis P (2020). Post-COVID-19 chronic symptoms: a postinfectious entity?. Clin Microbiol Infect.

[44] Ladds E, Rushforth A, Wieringa S, Taylor S, Rayner C, Husain L, Greenhalgh T (2020). Persistent symptoms after Covid-19: qualitative study of 114 "long Covid" patients and draft quality principles for services. BMC Health Serv Res.

[45] Assaf G, Davis H, McCorkell L, Wei H, O'Neill B, Akrami A (2020). An Analysis of the Prolonged COVID-19 Symptoms Survey by Patient-Led Research Team. Patient Led Research.

[46] Cirulli E, Schiabor BK, Riffle S, Bolze A, Neveux I, Dabe S, Grzymski JJ, Lu JT, Washington NL (2020). Long-term COVID-19 symptoms in a large unselected population. medRxiv.

[47] Sudre C, Murray B, Varsavsky T, Graham M, Penfold R, Bowyer R, Pujol JC, Klaser K, Antonelli M, Canas LS, Molteni E, Modat M, Jorge Cardoso M, May A, Ganesh S, Davies R, Nguyen LH, Drew DA, Astley CM, Joshi AD, Merino J, Tsereteli N, Fall T, Gomez MF, Duncan EL, Menni C, Williams FMK, Franks PW, Chan AT, Wolf J, Ourselin S, Spector T, Steves CJ (2021). Attributes and predictors of long COVID. Nat Med.

[48] Kang E, Lee SY, Kim MS, Jung H, Kim KH, Kim KN, Park HY, Lee YJ, Cho B, Sohn JH (2021). The psychological burden of COVID-19 stigma: evaluation of the mental health of isolated mild condition COVID-19 patients. J Korean Med Sci.

[49] What Is Posttraumatic Stress Disorder?. American Psychiatric Association.

[50] Xiao S, Luo D, Xiao Y (2020). Survivors of COVID-19 are at high risk of posttraumatic stress disorder. Glob Health Res Policy.

[51] Cooper J, Phelps AJ, Ng CH, Forbes D (2020). Diagnosis and treatment of post-traumatic stress disorder during the COVID-19 pandemic. Aust J Gen Pract.

[52] Andrade RMD (2020). A company doctor's role during the COVID-19 pandemic. Clinics (Sao Paulo).

[53] Spagnolo L, Vimercati L, Caputi A, Benevento M, De Maria L, Ferorelli D, Solarino B (2021). Role and tasks of the occupational physician during the COVID-19 pandemic. Medicina (Kaunas).

[54] Lewis C, Roberts NP, Bethell A, Robertson L, Bisson JI (2018). Internet-based cognitive and behavioural therapies for post-traumatic stress disorder (PTSD) in adults. Cochrane Database Syst Rev.

[55] Abrams EM, Shaker M, Oppenheimer J, Davis RS, Bukstein DA, Greenhawt M (2020). The challenges and opportunities for shared decision making highlighted by COVID-19. J Allergy Clin Immunol Pract.

