# Supplementary material for: The Impact of Long COVID-19 on Mental Health: Observational 6-Month Follow-Up Study
Source: JMIR Ment Health. 2022 Feb 24;9(2):e33704. doi: 10.2196/33704 (PMC8914795; doi:10.2196/33704)
Supplement: Multimedia Appendix 1 [file mental_v9i2e33704_app1.docx]

Respondents assessed for eligibility (n=2 159)

Excluded (n=220)

- Symptoms <21 days (n=14)
- Symptoms before 01/01/2020 (n=8)
- Respondents who were admitted to ICU (n=15)
- No gender available (n=9)
- No complete questionnaire (n=174)

Completed 1^st^ survey first week June (n=1 939)

- Test-diagnosed COVID-19 (n=421)
  - Hospitalized (n=102)
  - Non-hospitalized (n=309)
- Suspected COVID-19 (n= 1 518)

Completed 2nd survey first week Sept (n=1 005)

- Test-diagnosed COVID-19 (n=239)
  - Hospitalized (n=62)
  - Non-hospitalized (n=177)
- Suspected COVID-19 (n= 766)

No consent to be approached (n=383)

Did not respond to 2^nd^ survey (n=551)

Consented to be approached (n=1 556)

**Enrollment**

**Eligible**

**Follow-up**

**Multimedia Appendix 1.** Study flow chart
