# Supplementary material for: The Impact of Long COVID-19 on Mental Health: Observational 6-Month Follow-Up Study
Source: JMIR Ment Health. 2022 Feb 24;9(2):e33704. doi: 10.2196/33704 (PMC8914795; doi:10.2196/33704)
Supplement: Multimedia Appendix 2 [file mental_v9i2e33704_app2.docx]

**Multimedia Appendix 2.** Characteristics of patients with presumed COVID-19

|  | | **Patients with presumed COVID-19 (n=766)** |
| --- | --- | --- |
| **Women, n (%)** | | 652 (85.1) |
| **Age, years (median, interquartile range)** | | 48.0 (40.0-54.0) |
| **BMI, kg/m^2^ (median, interquartile range)** | | 24.7 (22.2-28.1) |
| **Married/living with partner, n (%)** | | 543 (70.9) |
| **Educational level, n (%)** | |  |
|  | Low | 19 (2.5) |
|  | Medium | 258 (33.7) |
|  | High | 484 (63.2) |
| **Pre-existing comorbidities, n (%)** | |  |
|  | None | 475 (62.0) |
|  | 1 | 196 (25.6) |
|  | ≥2 | 95 (12.4) |
| **Good self-reported health status before infection, n (%)** | |  |
|  | Before infection | 653 (85.2) |
|  | After 3 months | 33 (4.3) |
|  | After 6 months | 130 (17.0) |
| **Number of symptoms, median (interquartile range)** | |  |
|  | During infection | 14 (11-18) |
|  | After 3 months | 7 (4-9) |
|  | After 6 months | 6 (3-9) |

BMI, body mass index.
