# Supplementary material for: The Impact of Long COVID-19 on Mental Health: Observational 6-Month Follow-Up Study
Source: JMIR Ment Health. 2022 Feb 24;9(2):e33704. doi: 10.2196/33704 (PMC8914795; doi:10.2196/33704)
Supplement: Multimedia Appendix 3 [file mental_v9i2e33704_app3.docx]

a)

#

#

#

|  |
| --- |
|  |
| b)  *  * |
|  |

**Multimedia Appendix 3.** Percentage of (a) hospitalized and (b) non-hospitalized patients for separate Trauma Screening Questionnaire (TSQ) items three and six months after the onset of COVID-19 symptoms

*p≤0.05 3 months vs. 6 months

#p≤0.05 hospitalized vs. non-hospitalized patients
