# Supplementary material for: The Impact of Long COVID-19 on Mental Health: Observational 6-Month Follow-Up Study
Source: JMIR Ment Health. 2022 Feb 24;9(2):e33704. doi: 10.2196/33704 (PMC8914795; doi:10.2196/33704)
Supplement: Multimedia Appendix 4 [file mental_v9i2e33704_app4.docx]

**Multimedia Appendix 4.** Symptoms of post-traumatic stress disorder, anxiety and depression

|  | | **Patients with suspected**  **COVID-19 (n=766)** |
| --- | --- | --- |
| **Trauma Screening Questionnaire** | |  |
| Total score, points (mean (SD)) | |  |
|  | *After 3 months* | 4.3 (2.3) ^a^ |
|  | *After 6 months* | 3.9 (2.4) |
| Total score ≥6 points, n % | |  |
|  | *After 3 months* | 225 (29.4) ^a^ |
|  | *After 6 months* | 192 (25.1) |
| **HADS anxiety subscale** | |  |
| Total score, points (mean (SD)) | |  |
|  | *After 3 months* | 6.4 (3.9) ^a^ |
|  | *After 6 months* | 6.1 (4.0) |
| Total score ≥8 points, % | |  |
|  | *After 3 months* | 265 (34.6) |
|  | *After 6 months* | 256 (33.4) |
| **HADS depression subscale** | |  |
| Total score, points (mean (SD)) | |  |
|  | *After 3 months* | 7.4 (3.9) ^a^ |
|  | *After 6 months* | 6.4 (4.2) |
| Total score ≥8 points, % | |  |
|  | *After 3 months* | 359 (46.9) ^a^ |
|  | *After 6 months* | 301 (39.3) |

^a^ p≤0.05 vs. 6 months
