# Supplementary material for: The Impact of Long COVID-19 on Mental Health: Observational 6-Month Follow-Up Study
Source: JMIR Ment Health. 2022 Feb 24;9(2):e33704. doi: 10.2196/33704 (PMC8914795; doi:10.2196/33704)
Supplement: Multimedia Appendix 5 [file mental_v9i2e33704_app5.docx]

*

*

*

*

*

*

*

**Multimedia Appendix 5.** Percentage of patients for separate TSQ items three and six months after the onset of COVID-19 symptoms (patients with suspected COVID-19, n=766)

*p≤0.05 3 months vs. 6 months
