# Supplementary material for: The Impact of Long COVID-19 on Mental Health: Observational 6-Month Follow-Up Study
Source: JMIR Ment Health. 2022 Feb 24;9(2):e33704. doi: 10.2196/33704 (PMC8914795; doi:10.2196/33704)
Supplement: Multimedia Appendix 6 [file mental_v9i2e33704_app6.docx]

**Multimedia Appendix 6.** Self-reported pre-existing comorbidities

|  | **n=239** |
| --- | --- |
| I was not treated by a doctor before the corona infection, n (%) | 142 (59.4) |
| Anxiety, n (%) | 2 (0.8) |
| Arthrosis, rheumatoid arthritis, n (%) | 8 (3.3) |
| Burn-out, n (%) | 4 (1.7) |
| Cancer, n (%) | 2 (0.8) |
| Depression, n (%) | 5 (2.1) |
| Diabetes, n (%) | 2 (2.1) |
| Heart diseases, n (%) | 13 (5.4) |
| Hypertension, n (%) | 23 (9.6) |
| Kidney diseases, n (%) | 1 (0.4) |
| Lung diseases, n (%) | 39 (16.3) |
| Migraine, n (%) | 5 (2.1) |
| Obesity, n (%) | 6 (2.5) |
| Osteoporosis, n (%) | 3 (1.3) |
| Other, n (%) | 36 (15.1) |
| Parkinson’s disease, n (%) | 0 (0.0) |
| Stroke, n (%) | 0 (0.0) |
